# Building a Group-Based Opioid Treatment (GBOT) blueprint: a qualitative study delineating GBOT implementation

**DOI:** 10.1186/s13722-019-0176-y

**Published:** 2019-12-27

**Authors:** Randi Sokol, Mark Albanese, Aaronson Chew, Jessica Early, Ellie Grossman, David Roll, Greg Sawin, Dominic J. Wu, Zev Schuman-Olivier

**Affiliations:** 1 Malden Family Medicine Center, 195 Canal St, Malden, MA 02148 USA; 2 Outpatient Addiction Services, 26 Central St, Somerville, MA 02143 USA; 3 Somerville Hospital Primary Care, 236 Highland Avenue, Somerville, MA 02143 USA; 4 Revere Care Center, 454 Broadway, Revere, MA 02151 USA; 5 Center for Mindfulness and Compassion, 1035 Cambridge Street, Suite 21, Cambridge, MA 02141 USA

**Keywords:** Buprenorphine–naloxone, GBOT, Group visit, Shared medical appointment, Group psychotherapy, Implementation science

## Abstract

**Background:**

Group-Based Opioid Treatment (GBOT) has recently emerged as a mechanism for treating patients with opioid use disorder (OUD) in the outpatient setting. However, the more practical “how to” components of successfully delivering GBOT has received little attention in the medical literature, potentially limiting its widespread implementation and utilization. Building on a previous case series, this paper delineates the key components to implementing GBOT by asking: (a) What are the *core* components to GBOT implementation, and how are they defined? (b) What are the *malleable* components to GBOT implementation, and what conceptual framework should providers use in determining how to apply these components for effective delivery in their unique clinical environment?

**Methods:**

To create a blueprint delineating GBOT implementation, we integrated findings from a previously conducted and separately published systematic review of existing GBOT studies, conducted additional literature review, reviewed best practice recommendations and policies related to GBOT and organizational frameworks for implementing health systems change. We triangulated this data with a qualitative thematic analysis from 5 individual interviews and 2 focus groups representing leaders from 5 different GBOT programs across our institution to identify the key components to GBOT implementation, distinguish “core” and “malleable” components, and provide a conceptual framework for considering various options for implementing the malleable components.

**Results:**

We identified 6 core components to GBOT implementation that optimize clinical outcomes, comply with mandatory policies and regulations, ensure patient and staff safety, and promote sustainability in delivery. These included consistent group expectations, team-based approach to care, safe and confidential space, billing compliance, regular monitoring, and regular patient participation. We identified 14 malleable components and developed a novel conceptual framework that providers can apply when deciding how to employ each malleable component that considers empirical, theoretical and practical dimensions.

**Conclusion:**

While further research on the effectiveness of GBOT and its individual implementation components is needed, the blueprint outlined here provides an initial framework to help office-based opioid treatment sites implement a successful GBOT approach and hence potentially serve as future study sites to establish efficacy of the model. This blueprint can also be used to continuously monitor how components of GBOT influence treatment outcomes, providing an empirical framework for the ongoing process of refining implementation strategies.

## Background

Opioid use disorder (OUD) has reached epidemic proportions in the U.S. Every day, more than 115 people die after overdosing on opioids [1], and opioid overdoses recently surpassed motor vehicle accidents as the most common cause of accidental death [2, 3].

Fortunately, Medications for Opioid Use Disorder (MOUD), specifically methadone, buprenorphine–naloxone (B/N), and naltrexone, are highly effective [4]. After detoxification, patients without MOUD have a 90% chance of relapsing over the next year; with MOUD, 50–60% of patients remain in recovery, significantly reducing the risk of accidental overdose and death [5–7]. While methadone treatment is highly regulated and can only occur at specialized clinics, the passage of DATA (Drug Addiction Treatment Act) in 2000 enabled physicians to prescribe B/N in an outpatient setting [8], increasing access to and decreasing stigma of treatment.

While many outpatient providers currently offer B/N to patients via one-to-one provider visits, group-based opioid treatment (GBOT) [9] has recently emerged as a treatment option. In group visits, patients with similar chronic diseases (such as diabetes, hypertension, and obesity) are seen as a group in order to streamline provider efforts to review and meet health targets and provide education on their common health condition(s), increase access to care, enhance patient self-efficacy around lifestyle and behavioral changes [10], and increase self-management of chronic illness [11, 12]. Because patients living with chronic disease often feel a sense of shame and isolation, bringing individuals together in a protected and safe space can potentially create a healing community where members develop connections to others with similar conditions, learn from each other, and gain a sense of validation and hope [13].

In GBOT, patients struggling with OUD have the opportunity to support each other in a common goal of recovery, receive pharmacotherapy and behavioral counseling. GBOT can be delivered in two main formats: (1) Shared Medical Appointments (SMAs), in which B/N prescribing and group-based counseling all occur concurrently within the presence of the prescribing clinician, and (2) Group Psychotherapy, in which B/N prescribing occurs during individual patient appointments while group psychotherapy sessions can occur at a different day or time during the week [14].

Unfortunately, the GBOT model has not yet demonstrated consistent effectiveness, largely due to limited evidence. A recent systematic literature review assessing the efficacy, feasibility, and acceptability of a group-based approach to treating OUD found only ten peer-reviewed studies, which suggested feasibility even though it was markedly limited by study quality and size [15].

Despite its unproven effectiveness, the GBOT model holds the potential to benefit patients and providers. For patients, a group-based approach can create a sense of accountability, shared identity, and a supportive community unlikely to be achieved through individual visits with providers [9]. Patients prescribed B/N seeking group support may feel more accepted in a GBOT setting where medication is provided, unlike outside self-help groups, such as Narcotics Anonymous or Alcoholics Anonymous, where patients taking medication for OUD often report feeling judged or excluded and may not be viewed by others as abstinent while on maintenance medication [16, 17]. The GBOT model can also potentially increase access to both psychotherapeutic and pharmacological components of addiction care by coupling both approaches as opposed to requiring the patient to attend two separate appointments with two separate co-pays.

For providers, GBOT offers the potential to increase the number of patients being treated for OUD by enhancing the volume capacity among providers who prescribe B/N. For instance, in GBOT, primary care providers have the ability to see up to 32 patients in a typical 4-h primary care setting (if two GBOT groups are provided) [18]. By having a collaborative team-based approach to care that involves both prescribers and other providers (e.g., nurses, behavioral health, medical assistants, and administrative staff), the GBOT approach may mitigate provider burnout because they are no longer caring for this psychosocially complex patient population alone. It also utilizes the patients’ abilities to support each other, further taking the burden off the prescriber to problem-solve difficult addiction-related problems [18, 19]. Thus, the model may address concerns from physicians who obtain their B/N waiver, but do not go on to prescribe in a one-to-one setting, because of a perceived lack of time to see additional patients [20, 21], belief that reimbursement rates are insufficient [21], and limited access to mental health and psychosocial support [22]. Given its potential to benefit patients and providers, more widespread systematic implementation is needed to further assess its effectiveness as a delivery model on treatment outcomes, access to office-based opioid treatment, and provider behaviors. However, scant information exists on the more practical “how to” components of successfully delivering GBOT, and no systematic implementation algorithms exist, potentially limiting larger scale implementation and adequately powered scientific investigation. In a previous paper [14], we presented a case series to illustrate 4 variations of GBOT implementation across a large academic health system. All cases included B/N prescribing coupled with required office-based group counseling, in which the B/N prescribing occurred either at the same time or separately from the group counseling appointment.

In this paper, we hope to further delineate the key components to GBOT implementation by asking: (a) What are the *core* components to GBOT implementation, and how are they defined? (b) What are the *malleable* components to GBOT implementation, and what conceptual framework should providers use in determining how to apply these components for effective delivery in their unique clinical environment?

## Methods

We used the Standards for Reporting Qualitative Research (SRQR) reporting guideline checklist to ensure transparency and rigor in reporting our qualitative research (see Additional file 1: Appendix) [23].

### Population and setting

Cambridge Health Alliance (CHA) is an academic community health safety net system that serves over 140,000 patients in the metro-north Boston area with 13 primary care sites and 3 affiliated hospitals. Across this system, three OBOT (office-based opioid treatment) nurse case managers (RNCMs) [24] serve as the first point of contact for patients with OUD, triaging referrals, connecting them to services, and supporting and coordinating ongoing care once the patient has initiated treatment. Within this system, there are over one-hundred providers who are waivered to prescribe B/N. Currently, six outpatient sites provide B/N in a group visit format. Patients requiring a higher level of support services based on their addiction and/or psychiatric needs can be referred to the outpatient addiction services (OAS) intensive outpatient and integrated dual diagnosis treatment within our system. Those requiring an inpatient level of care, such as detoxification, long-term residential program, or partial hospitalization programs, are referred outside of the system.

### Study design

To create a blueprint delineating GBOT implementation, we reviewed literature about existing GBOT studies, best practice recommendations and policies related to GBOT, and organizational frameworks for implementing health systems change. We also conducted interviews and focus groups across 5 GBOT sites. After subsequent data analysis and triangulation of all this information, we identified key components to GBOT implementation, distinguished “core” and “malleable” components, and provided a conceptual framework for considering various options for implementing the malleable components.

### Data collection

Our team composed a general conceptual framing for the range of GBOT implementation components based on our previous research conducting a systematic literature review of GBOT models [15]. We then used this draft conceptual framing to develop the subsequent semi-structured interview guide (see appendix) that we employed during individual 1-h-long interviews with five individual GBOT site leaders. We later held two focus groups (average duration 75 min), which included seven of the GBOT site leaders representing all of the GBOT programs models [14] within our health system (including those participating in individual interviews), collecting perceptions of the framework and its components.

### Data analysis

We used a directed content analysis [25] approach in which our previous research [14, 15] and unanswered questions provided an overarching framework and categorical structuring through which to collect data (development of the interview guides) and analyze data.

For data we collected via individual interviews the first author and senior author independently coded our notes through iterative note comparison and subsequent discussions to arrive at twelve independent, individual codes related to GBOT implementation components that we then sorted into 5 “core” and 7 “malleable” components. We further revised the categories and their distinction as “core” vs. “malleable” through several processes: We held two focus groups with GBOT site leaders, we reviewed initial interview notes, we referred back to the 10 additional GBOT publications identified through our systematic literature review [15], and we used our personal experience as GBOT program directors to arrive at 6 core and 14 malleable components that characterize GBOT implementation. These final categories and the underlying rationale were then shared with the 7 GBOT site leaders, who independently reviewed them to validate their accuracy.

Once these 20 components of GBOT implementation were delineated, for each component, we then reviewed the related best practice policies and recommendations put forward by the American Society of Addiction Medicine (ASAM) [26–28], the National Institute for Drug Abuse (NIDA) [29], the Substance Abuse and Mental Health Services Administration (SAMHSA) [8, 30–33], and the Massachusetts Department of Public Health’s Bureau of Substance Abuse Services (BSAS) [34]. The senior author (ZSO) also identified key seminal papers that related to each GBOT component. Each co-author was assigned two GBOT components and reviewed these guidelines, related papers, and performed an independent PubMed search specific to their topics, summarized their findings, and these were then further reviewed and revised by the lead and senior authors (RS and ZSO).

While we reviewed content-specific literature focused on GBOT, at the same time, we also reviewed literature around organizational frameworks for creating and sustaining change within health care systems. Since our study is rooted in a case series approach, we considered organizational frameworks described in the case series literature which considers the contextual factors of structures, processes, and outcomes of systems-level changes [35–39]. This framing helped us identify GBOT implementation components. The lead and senior author (RS and ZSO) then created criteria to define “core” versus “malleable” components. When there was question or debate about this distinction, the authors compared components across the case series of GBOT models to test out the integrity of these definitions and sought further revisions from five GBOT case (site) leaders to arrive at the final six core and fourteen malleable components. Building off previous case series of translational research [40], we developed a novel framing to further describe the malleable GBOT implementation component options (presented in "Results" section), in which we triangulated data from literature review, best practices, policy, evidence, and our personal experiences.

Study quality was achieved through triangulation [41, 42] of data from multiple sources (literature reviews, best practices, policy, interviews, focus groups, and our personal clinical experiences). Through reflexive discussion [41, 42], the lead and senior authors (RS and ZSO, respectively) acknowledged their own leadership roles in GBOT implementation and potential to influence the results (development of an implementation blueprint) shaped by the model they helped create and that other research participants (the leaders of GBOT sites) are familiar with, in the same health system. To minimize this bias, the researchers first reviewed the empirical literature on GBOT implementation. They then utilized research assistants who had limited familiarity with GBOT to develop initial categories of implementation based on the current literature, create a semi-structured interview guide, and interview a working group of GBOT experts within the system. This process of using evidence and outsider perspective also guided the process for focus group data collection. Thus, the ongoing and iterative coding of GBOT implementation categories by the lead and senior authors was conducted in a way to minimize experimenter bias, in which the authors’ expectations could potentially increase the probability that the results occurred as predicted [43].

We did not seek Institutional Review Board (IRB) approval since this project was not considered human subjects research; rather, data was collected from co-researchers, all with expertise in GBOT implementation, and all participants in the working group are included as co-authors on this paper.

## Results

### Core components to GBOT implementation

The core components are foundational to GBOT implementation. We define these core features as those that are essential because they optimize clinical outcomes (those most frequently measured in clinical outcomes studies around OUD, including decreased opioid use and increased retention in treatment [15]), comply with mandatory policies and regulations, ensure patient and staff safety, and/or promote sustainability in delivery.

Based on a systematic review of GBOT models we previously conducted [15], focus groups, and interviews with GBOT providers across our healthcare system, we identified the 6 core components to GBOT implementation. See Table 1.

**Table 1:** Core components to GBOT implementation

1) Consistent application of expectations
Group expectations set through contracts and ground rules
Low tolerance for inappropriate patient behaviors
Low tolerance for substances of abuse (illicit and prescribed)
2) Team-based approach (medical assistant, front desk, nurse, B/N provider, psychologist)
3) Creating a safe and confidential space
4) Billing
Primary Care Provider
99213 if no individual appt. or
99214 if individual appt
Behavioral Health Provider
90853 for group psychotherapy or 96153 for health behavior code
5) Regular monitoring through drug screens and PDMP
6) Regular attendance and participation in groups

#### Consistent application of expectations

- Group expectations about attendance, substance use, and treatment-related behaviors are set through confidentiality agreements, group treatment agreements, and “ground rules” written and revised in collaboration with patients to promote safe interactions with appropriate boundaries. If violated, the terms are reviewed with the group or individuals. At some sites, some or all ground rules (e.g., importance of confidentiality) are read regardless of any violation, as a ritualistic way to start group.
- Sites promote low tolerance for inappropriate patient behaviors. For interpersonal behavioral issues, the patient is pulled aside and the behavior is addressed individually. For more serious issues, such as tampering with urine samples, displaying aggressive behavior, selling/sharing medication, or breaching patient confidentiality, patients are discharged from the GBOT program and referred to individual office-based opioid treatment (OBOT) providers within or outside our system.
- Sites promote low tolerance  for substances of abuse: Patients are discouraged from using prescription medications with abuse potential that are not prescribed to them (such as benzodiazepines, stimulants, gabapentin) and illicit drugs (alcohol, cocaine, heroin, etc.). They are expected to remain sober from non-prescribed medications and illicit drugs.  However, sites might respond in various ways when patients use substances of abuse (see “Malleable components to GBOT implementation” section).

##### Why this is core

Patients in group must understand the expectations of being part of a group and trust that each member is fairly treated and held to the same set of standards. Clearly defined rules with regular enforcement promotes honesty, transparency, and engagement. When expectations are not fair, unevenly or irregularly enforced, patients can feel resentment toward the group and/or group leaders, potentially causing them to become disengaged and ultimately resulting in attrition of group members [44, 45]. According to SAMHSA Treatment Improvement Protocol (TIP) 41, ‘a group member’s acceptance of the contract prior to entering a group has been described as the single most important factor contributing to the success of outpatient therapy groups’ [30].

#### Team-based approach to care

All sites use an interdisciplinary team-based approach to managing patient care. This may include a B/N prescribing provider (physician/physician assistant/nurse practitioner), a nurse with specialized training in addiction, behavioral health provider (e.g., social worker, psychologist, etc.), case manager, medical assistant, and/or front desk staff member(s). Each member has specific tasks and responsibilities for supporting the team. For example, designated front desk staff may check-in group patients separately from the main check-in line, creating a more streamlined process designed to avoid the stress of en-mass check-ins for other staff members. Prior to group starting, medical assistants can collect urine samples and distribute pre-visit questionnaires. Providers write the B/N prescriptions, bill the visits, provide medical management, and often facilitate group. Behavioral health providers can lead the psychotherapeutic skills component of group. Nurses can serve as case managers and as a patients’ first point of contact, check on patients who are struggling, and help connect patients to other levels of care. Case managers may help connect patients to support services, such as housing, transportation, or individual psychotherapy.

##### Why this is core

This approach promotes sustainability by supporting providers who manage patients with complex psychosocial co-morbidities, thus promoting optimal patient care while preventing provider burnout [46–48]. It is also consistent with the DATA 2000 buprenorphine waiver requirements, in which providers “have the *capacity* to refer patients for appropriate counseling and other appropriate ancillary services” when needed [32]. This team-based approach also aligns with the 2007 Joint Principles for Patient Centered Medical Home, which promote incorporating as many support services as possible within a primary care setting to promote care that is “personal, coordinated, continuous, and comprehensive” [49].

#### Creating a safe and confidential space

Patients should feel they are entering a physically and psychologically safe and confidential space where they can express themselves honestly and openly with the group and receive genuine reciprocal support. Thus, group leaders must promote therapeutic factors that breed healthy socialization behaviors between members.

##### Why this is core

Patients entering group treatment may harbor beliefs and behaviors that run counter to recovery goals. One-third of patients with substance use disorders (SUDs) have co-occurring serious mental health distress, and patients with SUDs (especially women) have substantially higher rates of trauma history than the average population [31]. External and self-stigmatization may prevent these patients from seeking treatment [50, 51]. Those who do may be wary of opening themselves up to others in a group setting. They may demonstrate maladaptive behaviors such as distrust, denial, intimidation, and shaming [52]. Participation in group-based treatment requires active listening and peer support where members provide feedback, confront harmful behaviors, and learn healthy coping skills from each other. Members must enter the group with a degree of vulnerability and be able to embrace honesty and openness [30, 52].

Group leaders must thus promote therapeutic factors that nourish healthy interactions between group members, creating an environment where patients can learn and grow from each other. Group leaders should promote the group therapeutic factor of universality (which challenges patients’ individual beliefs of being alone and that no one can understand their struggle). This helps to minimize stigma (external and self-imposed), invite vulnerability, and create opportunities for open, honest, and supportive interpersonal relationships [45]. Group leaders should also promote group cohesion (a sense of connectedness to other group members) by building a strong therapeutic alliance between the group leader and group participants. Group cohesion leads to increased group attendance, improves psychiatric and health outcomes [53–56], and yields higher levels of treatment engagement [57–61]. Specifically, accurate empathy and empathic listening supports the therapeutic alliance, accounting for 5% to 12% of the variance in substance use outcomes, whereas advice-giving from the group leader has been associated with poorer outcomes [55]. Because the emotional connections forged between group members, the group leader, and the group as a whole are considered crucial to promoting optimal patient outcomes, effective group leaders should demonstrate a strong sense of self-assurance, expertise, creativity and flexibility, and utilize humor and levity when appropriate [29, 30, 58].

#### Billing

Across the system, we follow Center for Medicaid and Medicare Services (CMS) regulations and standard rules in billing using Evaluation and Management (E/M) coding for the Shared Medical Appointments or group psychotherapy codes for behavioral health provider group visits.

Currently, there are no nationally set standards or explicit guidelines for coding and billing for group visits. Medicare has issued a statement regarding group visits: “Under existing CPT codes and Medicare rules, a physician could furnish a medically necessary face-to-face E/M visit (CPT code 99213 or similar code depending on level of complexity) to a patient that is observed by other patients. From a payment perspective, there is no prohibition on group members observing while a physician provides a service to another beneficiary” [10].

Thus, group visits can be billed as follows: [10].

1. If the group type is provided by a physician, physician assistant (PA), or nurse practitioner (NP) and there is documentation of: a) individual medical evaluation and management and b) care provided in the context of a group visit, it can be billed using a medical E&M code 99213/99214, based on appropriate level of medical complexity. Under this code, there is no limit on the number of patients that can be included; it can be billed in the presence or absence of another provider. In practice, we find that 16 patients is the upper limit for a GBOT group to allow adequate time for individual medical evaluation and management in a group context, though there is no specific regulation specifying this as limit.
2. If the group is co-led or run solely by a licensed behavioral health providers (social worker, psychologist, etc.), it can be billed in one of two ways:
  - 90853 (this is a group psychotherapy code); Under this code, the group:
    Can be led by any licensed behavioral health provider.
    Typically lasts 45–60 min and time should be documented*(though there is no specific requirement for the amount of time spent on any specific psychotherapeutic component).
    Cannot be billed for time that overlaps with E/M billing, requiring solely group psychotherapy to last for a 50-min time period, though group psychotherapy can follow or precede E/M visits.
    Psychotherapy must be documented for a psychiatric condition.
    Capacity is limited to 10–12 patients based on Medicaid/Medicare rules* and the number of patients in group must be documented.

*Since Medicaid and Medicare have slightly different rules and these can vary by state, and whether the group visit is occurring in a hospital or federally-*qualified health center, we recommend practitioners check with their state Medicaid/Medicare offices for group capacity and duration of group psychotherapy requirements.*

b. 96153 (this is a “health behavior code”); Under this code, the group:
  Must be led by a clinical psychologist (at Ph.D. level).
  Must be linked to medical (not psychiatric) diagnosis.
  There is no group capacity limit.

##### Co-billing

If the group is co-led by a physician/physician assistant/nurse practitioner (who provides individualized medical management) and a behavioral health provider (who facilitates group discussion related to behavior change and mental health) and each provider documents separate clinical notes, the visit can be billed as *both* an E&M code (99213/99214 from the physician/physician assistant/nurse practitioner) *and* with the behavior health code (96153) from the licensed behavioral health care provider as long as the above conditions are met. CMS guidelines do not specifically mention the ability to bill a group visit with both the 99213/99214 (medical management) and 90853 (group psychotherapy) CPT codes. Thus, we recommend checking with your health system’s billing department for further guidance on billing both of these codes; and, if the 90853 code is used (with or without 99213/99214), we recommend ensuring the group includes at least 50 min of the psychotherapy component preceded or followed by adequate time for E/M visits.

##### Why this is core

Billing correctly is important to comply with institutional, state, and federal requirements. Additionally, because reimbursement rates are tied to billing, correct billing ensures that group delivery of care remains sustainable for clinical operations.

#### Regular monitoring through toxicology screening and prescription monitoring

Across all sites, regular toxicology testing occurs, though the frequency of testing and whether or not testing is tied directly to the group visit varies (see malleable component).

##### Why this is core

Toxicology testing supports patient safety through detection and deterrence of substance use. The American Society of Addiction Medicine recommends using drug testing as a tool supporting recovery rather than enacting punishment [28]. There is no agreed upon standard regarding the frequency or duration of testing. Some studies have suggested gathering a baseline urine drug screen prior to initiation of buprenorphine [62–64] and some form of continued drug monitoring for overall compliance. Additionally, the use of random (compared to scheduled) drug screens has shown significant reductions in illicit drug use and is recommended as a best practice [26, 30]. It is recommended to set the frequency of testing higher at the start of treatment, when patients are known to more frequently engage in continued drug use [26]. While sites may screen with different frequencies, each site should ideally adopt a universal approach for all patients so patients feel they are being monitored fairly. Checking the state’s prescription drug monitoring program also helps ensure patients are taking controlled substances appropriately and is required in many states.

#### Regular attendance and participation in groups

All groups have set schedules, occurring weekly, every other week, or monthly at specific days and times. To promote group cohesion, patients are generally expected to attend regularly.

During each group visit, patients are given the opportunity to “check in” about their recovery status and general wellbeing. Additional substance abuse-related educational content is provided for the group and takes various forms (see “Malleable components to GBOT implementation” section). For the SMA approach, the “check in” is considered mandatory since this format does not include an encounter with an individual provider. In some groups, patients also complete pre-visit questionnaires, allowing them to disclose substance-related behaviors or other medical/psychiatric issues needing attention that they do not feel comfortable disclosing in the group. For the group psychotherapy approach paired with individual prescribing sessions, sharing is encouraged but is not required to occur in group (patients can ‘pass’) since they also have the opportunity to “check in” with the provider individually.

##### Why this is core

Regular attendance and participation in group sessions have been shown to support recovery. The act of regularly showing up on time, being accountable to a group of people, and giving and receiving feedback and support from others struggling with similar problems is known to provide therapeutic value thus embodying the key features that define a group-based approach [29].

Recently several innovative bridge clinic models have been developed offering low threshold daily drop-in prescribing groups [65, 66] for the purposes of harm reduction and/or linking inpatient units to outpatient treatment with the composition of the group varying from day to day. These models are helpful for initial accessibility but may not be designed to facilitate development of group cohesion to support stability and medication maintenance over time, and attendance may vary widely from day to day and week to week. For that reason, we do not include this drop-in prescribing group format within the proposed GBOT model, though we do believe it holds promise as a way to initially engage patients and then link them into longer-term opioid treatment with B/N.

### Malleable components to GBOT implementation

The malleable components create flexibility in the GBOT model to optimally support each unique context and setting.

#### Based on a systematic review of various

GBOT models and focus groups and interviews we conducted with GBOT providers across our healthcare system, we identified fourteen malleable components to GBOT implementation. See Table 2.

**Table 2:** Malleable components to GBOT implementation

Malleable component	Options
I. Approach to slips and lapses	Approaches range from abstinence-only to relapse prevention to harm reduction (see below for further details)
II. Where and how buprenorphine prescription distribution occurs	Prescriptions can be distributed in or outside of the group session. Prescriptions may be provided as paper copies or e-prescribed directly to pharmacy. Patients may be required to attend every group or a certain percentage of groups/month in order to get a prescription
III. Whether or not individual appointments are offered in association with GBOT	Group patients may be offered individual appointments associated with the group visit before or after the group session on an as needed or scheduled basis. Alternatively, individual appointments may not be associated with the group session, and the patient is encouraged to schedule a separate appointment with an onsite provider, such as their primary care provider or psychiatrist
IV. Mix of patients based on status in recovery	Patients may attend groups with others in similar stages of recovery (“leveled groups”) [67] in which they graduate from one level to enter a different level (weekly, bi-weekly or monthly group). Or they may attend with patients in various stages of recovery (“mixed groups”)
V. Mix of patients based on other factors (SUD/psychiatric diagnoses, MAT, gender, and other identities): homogenous versus heterogeneous groups	Some groups may be exclusively for patients with OUD on B/N or XR-naltrexone, while other groups may include patients with non-OUD substance use disorders, such as alcohol use disorder (AUD). Some groups are for people with certain types of psychiatric symptoms, e.g., bipolar [68], PTSD [69] groups. Some groups are gender specific [70] and others are tailored to other group identities, such as LGBTQ or ethnicity, since having a shared identity and background has demonstrated improved SUD-related outcomes [71–76]
VI. Type of provider facilitating group, their associated background, training, and skill set	Groups may be facilitated by primary care providers (physicians, physician assistants, nurse practitioners) and/or licensed behavioral health care providers (social workers, certified addiction registered nurses, psychologists).
VII. Psycho-educational approach	Based on their experience and background training and the needs of the group participants, facilitators can run various types and mixes of psycho-educational approaches: support, cognitive behavioral therapy, educational, skills-based, and interpersonal processing groups (see below for further details)
VIII. Buprenorphine dosing	Dosing typically ranges between 2–24 mg buprenorphine/day (see below for further details)
IX. Duration and frequency of groups	Group visits can last from 30 to 120 min. They can be offered several times a week, such as in an intensive outpatient setting or less frequently in other settings
X. Enrollment scheduling and size of group	Group visits can enroll patients through various practices, including fixed membership (with a stable cohort of participants over time) or rolling admissions (with new members continuously entering and leaving the group). Groups can also use partial rolling admission (with a new cohort entering together every 4 weeks) to minimize impact on group cohesion [77, 78] Participation may be time-limited, with a defined number of sessions or a defined curriculum, or it may be ongoing, lasting until members meet specific goals, such as reduced drug use or stabilization of medical and mental health issues. or last indefinitely Size of groups can range from 9 to 16 patients (based on best practice recommendations [15]) but are often smaller due to patient absences and CMS billing restrictions for group psychotherapy. Group size might be limited by B/N-prescriber’s capacity [8]
XI. Admissions process	Patients can enter GBOT programs either without recent prior addiction treatment or after completing a more intense program of recovery. All patients have individual appointments with B/N-prescribing providers before beginning group-based treatment
XII. Inductions and observed dosing	Sites can offer in-office inductions or home induction with phone call follow-up based on patient desires, medical necessity, and provider safety concerns [79–83]
XIII. Contingency management	Sites may or may not offer rewards (positive reinforcement) or remove negative stimuli (negative reinforcement) in response to patient behaviors. For example, sites may reward patients doing well (which can be variably defined based on criteria such as attendance rates, toxicology results, social/functional outcomes) by spacing them out to less frequent groups [82, 83]. Some programs may also use monetary rewards to encourage attendance or abstinence [84, 85], using direct payments or a fishbowl system [86, 87]. The rewards can be removed when people do not meet the agreed upon expectations. Contingency management works best when the rewards are given at a high frequency rate for small, manageable behaviors and occur as close in time to the targeted behavior as possible (for example, during group right after each toxicology result rather than after a month of toxicology results) [62]
XIV. Monitoring for illicit and non-prescribed drug use and diversion: type of screening test and frequency of screening	All sites should monitor for drug use. However, the type of screening and frequency can vary: Sites can use urine screens, oral fluid swabs, and/or pill counts to monitor illicit and non-prescribed drug use and diversion. These can be employed regularly at every group visit and/or patients can be called to come in randomly in between group visits or both. The tests can be employed at the group visit itself and/or at individual appointments associated with the group, based on attendance frequency expected for each patient

### Determining how to consider applying each of the malleable components

To help providers choose the best malleable components for their practice, our team developed a conceptual framework comprised of three dimensions:

1. Empirical considerations: Is there *scientific evidence, best practices or expert opinion* that can guide my choice?
2. Theoretical considerations: Are there underlying *philosophical* or *psychological* considerations that best align with my current practice setting and culture? Are there recommendations or guidelines from patient populations with SUD in general that can be applied to patients with OUD?
3. Practical considerations: Does the *environment* or *context* in which I practice (at the systemic, group, or individual levels) support specific logistical and operational components over others?

### Examples of application of malleable components

Below are three examples of how a site might consider employing a malleable GBOT component.

*Example 1: Approach to ongoing non*-*opioid substance use and/or relapse* When patients enrolled in GBOT programs on B/N continue using or return to using illicit substances, GBOT clinicians must determine how they will respond since their responses ultimately impact the culture of the group and hence the well-being of all group members. Options range from a strict abstinence-only policy [88] to a harm reduction approach [89]. A moderate approach is relapse prevention, with the goal of managing slips^1^ and lapses^2^ in order to prevent full blown relapses^3^ [90, 91].

#### Empirical considerations

Based on the American Society of Addiction Medicine (ASAM) review of the literature, there is a paucity of evidence examining the preclusion of OUD treatment among OUD patients who continue to use other psychoactive substances [27]. Evidence demonstrates that patients who actively use cocaine at intake to OUD treatment with B/N have lower treatment retention [92] at 3 months. Also, the frequency of opioid, amphetamine, cocaine and cannabinoid use during treatment may be associated with shorter periods of treatment retention during 6 months of treatment [93, 94]. Yet, the evidence is mixed regarding the effects of benzodiazepines [95] and marijuana [96] use, which are not always found to have a harmful effect on B/N treatment retention. Overall, patients who are able to engage in abstinence-only behaviors are less likely to relapse and more likely to remain in treatment, though further research is needed to assess the impact of benzodiazepine and marijuana use.

The National Institute on Drug Abuse, public health departments, and specialty professional organizations recognize OUD as a chronic disease in which lapse is common and an expected part of care [29, 34]. Discontinuing or tapering B/N increases the risk of opioid relapse and the rate of program attrition. Numerous studies have demonstrated that, compared to patients with OUD tapered with B/N, those maintained on B/N had significantly better outcomes related to relapse [97–99], retention rates [97, 100] and craving scores [99]. While these studies did not specifically assess patients who were enrolled in a B/N program and relapsed on illicit substances, they imply that patients who are not maintained on medication for MOUD have poorer outcomes than those who are maintained. Thus, programs that focus on relapse prevention and harm reduction and continue to prescribe B/N despite lapse to substance use may support patients who do not do well with abstinence-only approaches. GBOT providers might thus see benefits in continuing patients on B/N—despite relapse on substances—to prevent relapse on opioids, overdose, and death [89, 90].

#### Theoretical considerations

When deciding how to respond to group members’ substance use, GBOT providers might grapple with supporting individual patients versus protecting the group, the safety of others in the group trying to remain abstinent, and the group’s associated norms and culture [17].

Some GBOT providers may ascribe to the belief that all group members should adhere to the ground rule that use of opioids and other substances is a relapse; one-time or multiple violations of this rule may mean that a patient cannot adhere to group expectations and requires a higher level of care or non-group-based care. They may believe it is in the group’s best interest to adhere to this abstinence-only policy. There is substantial anecdotal evidence that when patients using illicit substances join and continue to participate in an otherwise abstinent SUD treatment group, they can trigger or invite others to slip, lapse or relapse. Yet, since OUD is a chronic condition [101], often characterized by periods of relapse and recovery, abstinence-only paradigms may run the risk of alienating patients with more severe disease or other social and structural burdens.

Thus, providers must consider how best to promote abstinence when group members are at various stages of recovery and readiness. Some providers utilize a 24-h rule in which group members who have used illicit drugs during the past 24 h are precluded from group, allowing the group to remain a safe space for those who are abstinent. Others might continue the group treatment but enhance support, such as individual psychotherapy appointments or twelve-step meetings. They attempt to balance the abstinence-only norms of group with the individual patient’s desires and capabilities. Since the data on treatment harms related to marijuana and benzodiazepine use during B/N treatment are not as consistent as with cocaine and opioids, some groups might allow patients to remain in group for benzodiazepine slips or marijuana use (especially in states where it is legalized or medically prescribed) when they might otherwise preclude someone from a group who is using cocaine or opioids. However, providers should ensure treatment ground rules are consistent and the impact of substance use on ongoing participation in group-based treatment is not arbitrary; otherwise, inconsistent application of expectations and associated consequences could lead to resentment and confusion among group participants.

Still other providers may ascribe to a full harm-reduction approach. Recognizing that patients who relapse might not opt for a higher level of care, providers may choose to keep the patients in group, believing that this approach offers the patients a better chance of recovery than discharge from group. Regardless of what theoretical approach providers choose to take, it is imperative that providers carefully monitor whether one patient’s substance use puts other group members at risk, remembering the maxim that we first do no harm.

#### Practical considerations

Providers’ responses to substance use might be guided by practical considerations, such as access to care. Providers with limited slots for group member attendance may gravitate toward an abstinence-only policy, reserving patient slots for those exhibiting improvement with group treatment, as evidenced by abstinence-only behaviors, and referring patients who are struggling to higher levels of care. Other providers might have greater capacity to support patients who are struggling in their recovery and keep them in group.

*Example 2: Buprenorphine dosing* When prescribing B/N for individual patients in a group setting, providers must decide the dose for each patient, if they will set a dosing limit for the group, and if so, what that limit will be.

#### Empirical considerations

Evidence suggests that most patients stabilize and are less likely to relapse at doses of buprenorphine between 12 and 16 mg/day, though some patients may require up to 24 mg/day to provide full mu-opioid receptor blockade necessary to prevent withdrawals and cravings [102–105]. Providers can mitigate withdrawal symptoms beginning at a 2 mg dose and provide opioid blockade at 16 mg for most patients using heroin or oxycodone [104]. Patients potentially needing higher doses to achieve blockade include those using increasingly potent illicit opioids (such as synthetic fentanyl analogs) [102], those with a history of more severe drug use [106], pregnant patients, [107, 108] and those with more severe pain [109]. A recent meta-analysis found that higher buprenorphine dose (16–32 mg/day) enhances retention and reduces opioid use [110].

Because buprenorphine has a half-life of 36 h [111, 112], once daily dosing should provide adequate opioid blockade. Lower dosing in more frequent intervals (such as BID, TID, or QID dosing) can cause fluctuating withdrawal symptoms and increase stress response [113], making patients more susceptible to stress-induced craving during the day [114] and potentially compromising their recovery. Because buprenorphine has a ceiling effect for respiratory depression, providers should choose a high enough once-daily dosing to achieve opioid blockade to reduce the possibility of overdose [115]. In a few specific special physiologic situations, patients may benefit from split dosing. For instance, pregnant patients may require greater and more frequent dosing (three or four times/day) to minimize withdrawal and improve adherence due to increased plasma volume [107, 108]. Because the therapeutic effect of buprenorphine on spinal cord opioid receptors involved in pain transmission is shorter (6–8 h) than it is on brain opioid receptors involved in opioid withdrawal (24 h) [115], patients with chronic pain may also benefit from split dosing [116] or additional small doses later in the day (e.g., 16 mg qAM, 4 mg qPM, 4 mg qHS).

#### Theoretical considerations

Numerous theoretical considerations impact a GBOT clinician’s decision about a group dosing limit, including the impact on group members, intention for minimal necessary dose, concerns about diversion [117, 118], and encouragement of once daily dosing [108, 112, 115].

When setting a lower prescribing limit, some GBOT providers may decide to prescribe the minimum dose necessary to stave off craving and withdrawal, even if that is less than the amount (12–16 mg) shown to be beneficial in most studies, attempting to limit overprescribing or potential diversion. Other providers may encourage patients to take the evidence-based therapeutic dose, even when patients request to be on lower doses, attempting to maximize likelihood of success in their recovery.

Some GBOT providers choose to set an upper prescribing limit for all patients in groups, such as 16 mg/day to prevent everyone in the group from asking for higher and higher doses and to mitigate concern for diversion. Yet, for those who may benefit from a higher dose (16–32 mg), a group dosing limit could reduce their potential for recovery. In addition, some providers are more concerned about diversion risk and others appear to be less worried. Since the strongest predictor of people using diverted B/N is inability to access legal B/N treatment [119] and most people are using it to prevent withdrawal or for self-treatment [118], many providers hold the belief that diverted B/N leads to less people overdosing at the community level [117, 118]. In contrast, other providers are more concerned about their prescriptions going to others for whom they did not intend to prescribe and the legal risks that widespread diversion could have on their medical licenses. No matter one’s belief, providers must adhere to DEA guidelines for diversion prevention and control strategies (e.g. pill counts and prescription monitoring program checks) [29], and group programs may feel more comfortable practicing without an upper dosing limit for the group if they implement strong diversion prevention and control strategies.

Providers should also encourage once daily dosing so that patients treat B/N like any other daily standing medication instead of taking it multiple times as needed throughout the day in response to life stressors or their mood.

#### Practical considerations

Regardless of providers’ philosophy, their dosing decisions may ultimately be influenced by more practical day-to-day considerations, such as paperwork and time constraints. For example, many states and insurance payers require prior authorization requests for doses of buprenorphine higher than 16 mg/day [120].

*Example 3: Psychotherapeutic, behavioral, and educational approaches* In selecting content for GBOT, there are several types of substance use disorder groups commonly employed: Educational [33, 121], Skills development [33, 121], Cognitive Behavioral Therapy (CBT) [33, 121], Dialectical Behavioral Therapy (DBT) [122, 123], Acceptance Commitment Therapy (ACT) [124, 125], Support [33, 121], Mindfulness [12, 126], 12-Step facilitation [127, 128], Interpersonal process groups [33, 121],Contingency Management [129], Community Reinforcement [130], and other specialized groups (such as trauma or anger management) [33]. In practice, most substance use disorder groups offer a mix of these approaches—an eclectic approach [33].

#### Empirical considerations

Since most of the current evidence has focused on the effectiveness of psycho-educational approaches for patients with substance use disorders in general, there is limited empirical evidence specifically on OUD-based patient populations. Clinical trials studying various behavioral approaches in managing OUD concluded that individual counseling does not have a consistent impact above high-quality standardized medication management approaches [131]. Some evidence has demonstrated that, among patients with OUD on MOUD, contingency management approaches significantly improve retention in treatment [132–135] and relapse rates on opioids [134–136] and other substances [117, 136–143]. However, while CM has some proven benefit in preventing opioid use at the individual level, its benefit in preventing opioid use in a group setting has not been demonstrated.

#### Theoretical considerations

A review of various psychotherapeutic modalities for SUDs (not OUD specifically) found that some treatments provide more benefit than others for specific subpopulations, but no treatment is consistently superior to others in all patient populations [144].

Providers may choose to employ specific therapeutic modalities based on the patient population. For example, among patients with low-psychiatric symptoms, 12-Step Facilitation Therapy displays the highest abstinence rates compared to Cognitive Behavioral Therapy and Motivational Enhancement Therapy [145]. However, all three treatment modalities perform poorly among patients with high-psychiatric symptoms [146]. Motivational Enhancement Therapy is most effective for patients with higher levels of anger while CBT and 12-Step Facilitation are most effective with patients with lower levels of anger [126]. Among female patients with SUD and borderline personality disorder, Dialectical Behavioral Therapy (DBT) may be most effective [147–149].

Providers may also consider the patients’ stage of recovery in selecting their psychotherapeutic approach. For example, patients in early recovery might respond better to educational, motivational, and support group approaches, while those in later recovery might respond better to CBT or interpersonal processing groups [29, 30]. Despite the lack of evidence supporting specific psychotherapeutic ingredients, common factors across all psychotherapeutic group interventions (previously described in “Core components to GBOT implementation” section) remain crucial to successful group outcomes.

#### Practical considerations

Given the limited supply of behavioral health professionals and other providers with advanced psychological training, GBOT sites will likely choose specific psychological content based on the skills of the available personnel. For example, facilitating a support or educational group often requires less sophisticated training than facilitating a skills-based, CBT, mindfulness, or interpersonal processing group. Additionally, since most GBOT sites will care for a heterogeneous patient population with a mix of co-morbid mental health diagnoses, providers will likely not try to tailor their psychological content to a single patient population to avoid limiting access to care.

## Discussion

For providers looking to provide group-based opioid treatment (GBOT) to patients with OUD, we provide a blueprint for implementation that identifies and describes 20 key components to implementation. Using organizational frameworks around implementing health care system change, we identify 6 “core” components based on the essential foundations of optimizing clinical outcomes, complying with mandatory policies and regulations, ensuring patient and staff safety, and promoting sustainability in delivery. These include: consistency in setting group expectations around norms and ground rules and responding to aberrant behavior and relapses; taking a team-based approach; ensuring a physically and psychologically safe and confidential space; billing based on Center for Medicare and Medicaid Services (CMS) rules and regulations; regular monitoring; and ensuring regular patient participation. We also have identified 14 malleable components to GBOT implementation and provide a novel conceptual framework that providers can apply when deciding how to employ each malleable component that considers empirical, theoretical and practical dimensions.

While GBOT is a relatively new delivery model for treating OUD, a previous systemic review suggests it is a feasible option for care delivery though its efficacy and acceptability remain to be proven [15]. GBOT may provide enhanced patient [9] and provider benefit [20–22] compared to traditional one-on-one patient-provider visits. This paper builds on our prior case series of GBOT models across a large academic health system [14], and it is the first to delineate a framework for organizing the components involved in implementing GBOT. Development of this framework creates the opportunity to measure each component of a site’s GBOT program, thus providing a roadmap for ongoing research about the impact each malleable component may have on GBOT effectiveness.

Given the need to develop effective implementation strategies for treating OUD, the GBOT delivery model may serve as a venue to increase access to care by providing care to large numbers of patients at non-stigmatizing, easily accessible outpatient sites, coupling prescribing of evidence-based medication for OUD with psychotherapeutic and psychosocial support, while providing comprehensive services that help patients in meeting their behavioral health and primary care needs. Additionally, this model may be more easily applied in primary care sites that already utilize a team-based approach to providing group visits for other chronic diseases, though further training may be needed to specifically support patients with OUD. The blueprint we outline can support sites in developing the logistical and operational components needed to implement group visits for treating OUD while also highlighting implementation considerations that are specific to this patient population.

### Limitations

Because GBOT is a relatively new mechanism for treating OUD, there is currently little empirical data (with only 10 GBOT models identified in a previously published systematic literature review) to develop a comprehensive understanding of GBOT implementation. Additionally, many recommendations and policies related to the components of GBOT implementation have been informed by best practices or expert opinion rather than rigorous randomized controlled trials or comparative effectiveness studies. Additionally, our findings build off a case series with inherent methodological limitations: thematic saturation may not have been achieved for each component due to the use of only 7 GBOT providers; data collection depended on note-taking and not recordings; and GBOT implementation occurred in a unique environment, which is a single academic health system with robust primary care behavioral health integration and team-based support and resources in urban and suburban settings. Thus, while we sought to comprehensively triangulate multiple types of data, the generalizability of our findings may be limited.

### Future directions

Since most of the 20 core and malleable components of GBOT implementation we identified currently have no robust evidence to support their utilization, the blueprint we provide here is meant to be a starting point. Knowing what evidence does exist can allow providers to incorporate evidence-based practices into their implementation. Future research is needed to further conceptualize these components, identify any additional components if they exist, and build a more robust understanding of how to individually tailor each malleable component, based on a site’s unique contextual factors, to produce successful GBOT implementation. We are developing a GBOT manual that outlines the empirical, theoretical, and practical considerations for each malleable component. We will apply this approach as we consult with other sites in implementing the GBOT model and iteratively revise and augment the manual based on lessons learned across GBOT sites. We are also developing and testing a fidelity scale to assess and rate how rigorously a site encompasses this evidence-based GBOT model.

As the number of GBOT sites grow, we hope to learn from other researchers who explore the evidence base as well as the theoretical and practical considerations related to the key components of implementation we have identified. We hope that the blueprint we have provided in this paper will serve as an initial framework from which to build a richer, more in-depth understanding of how to successfully implement the GBOT model to treat patients with OUD in the outpatient setting.

## Conclusion

Outpatient prescribers looking to administer B/N for the treatment of OUD via a group visit approach (GBOT) should consider the six core features that comply with mandatory policies and regulations, promote safety, sustainability in delivery, and optimal clinical outcomes. They should also recognize 14 malleable implementation components and ask categorical questions related to empirical, theoretical, and practical dimensions to determine how to apply various choice points at their specific site. While further research of the efficacy of GBOT and its individual implementation components is needed, the blueprint outlined here provides an initial framework to help outpatient sites implement a GBOT approach and hence potentially serve as future study sites to establish efficacy of the model. This blueprint can also be used to continuously monitor how components of GBOT influence treatment outcomes, providing an empirical framework for the ongoing process of refining implementation strategies.

## Supplementary information

**Additional file 1.**

## Data Availability

Data sharing is not applicable to this article as no datasets were generated or analyzed during the current study.

## Abbreviations

ACT: acceptance commitment therapy
ASAM: American Society of Addiction Medicine
B/N: buprenorphine–naloxone
BID: twice a day
CBT: cognitive behavioral therapy
CHA: Cambridge Health Alliance
CM: contingency management
CMS: Centers for Medicare and Medicaid Services
CPT: Current Procedural Terminology
DATA: Drug Addiction Treatment Act
DBT: dialectical behavioral therapy
DEA: Drug Enforcement Administration
E/M: evaluation and monitoring
GBOT: group-based opioid treatment
MOUD: Medications for Opioid Use Disorder
OAS: Outpatient Addiction Services
OBOT: office-based opioid treatment
OUD: opioid use disorder
RNCM: nurse case manager
SAMHSA: Substance Abuse and Mental Health Services Administration
SMA: shared medical appointment
SRQR: Standards for Reporting Quality Research
SUD: substance use disorder
TID: three times a day
TIP: Treatment Improvement Protocol
QID: four times a day

## Footnotes

1 An impulsive action that happens once with regret.

2 A return to the addiction behavior that is time-limited with less immediate insight.

3 A return to original level of use or problematic behaviors.
