# Supplementary material for: Building a Group-Based Opioid Treatment (GBOT) blueprint: a qualitative study delineating GBOT implementation
Source: Addict Sci Clin Pract. 2019 Dec 27;14:47. doi: 10.1186/s13722-019-0176-y (PMC6935085; doi:10.1186/s13722-019-0176-y)
Supplement: Supplementary file 1 — Additional file 1. [file 13722_2019_176_MOESM1_ESM.docx]

**Appendix**

**I.OBOT Interview Guide**

**Introductions**

Briefly describe who we are

Do you mind introducing yourself and what you do at CHA?

**General**

1.       Who runs the sessions? What degrees do they have and what are their roles at Cambridge Health Alliance?

2.       Besides the group leader, who else helps with the group?

3.       What is the length of group sessions?

4.       How frequently do they meet?

5.       How are you billing sessions?

6.       Do you have a group size cap or a limit on how many people you can have in one room at a time?

7.       How many patients are currently in your group?

8.       What is the consistency of your group leaders? Do you go back and forth between leaders?

9.       How do you manage vacation and who covers the group?

**Buprenorphine**

10.   How much buprenorphine are most of your patients on?

11.   Do your patients take buprenorphine once a day or more than once a day?

12.   When are prescriptions written? Are they written in, before, or after group?

13.   How do you incorporate urine testing into your workflow?

14.   Is there anything else you do besides urine tests to monitor patients?

**Patients**

15.   What are the inclusion/exclusion criteria for starting group?

·         Co-morbid psych diagnoses

·         Co-dependency

·         On any problematic medications, including benzodiazepines, gabapentin, Adderall, ETOH, other

·         Age

16.   Where is the location of your group?

17.   Do some patients come more frequently than others or is it a set group every time?

18.   How do you determine how frequently your patients come to session?

19.   Do you ever have drop-ins or is it a set invitation?

20.   Who else is present or welcome in the group outside of the patients?

21.   How often do you bring new patients into group?

22.   How do people get referred to group? Do they have to meet with an individual doctor before starting group?

23.   Do patients stay in the same group or change groups over time?

24.   Is there staging, such as introductory or second-level groups?

**Didactics**

25.   How much time does each person get to do their individual check-in?

26.   What do they talk about during their individual check-ins?

27.   Do you use any of these (yes/no):

·         Cognitive Behavioral Therapy

·         Motivational Interviewing

·         Dialectical Behavioral Therapy

·         Matrix model

·         Supportive counseling

·         12-step facilitation/AA or NA

·         Contingency management (if so, what kind)

·         Medication management group

·         Nutrition

·         Stress reduction

·         Other

28.   Could you describe content of your sessions and the didactic and psycho-educational components?

29.   Is there a component of private, individual treatment incorporated into your group? If so, what level of individual treatments is available to people in group?

a.       *Is it ad hoc after group, is it ‘peri’ group check-ins (step out of group, do their individual check-ins, then step back in) is it weekly?*

30.   Is there cross-talk between patients in the group?

31.   Do you incorporate management of other co-morbid diseases frequently seen in patients with drug addictions such as HIV, or Hepatitis C?

32.   Do you use a manual to guide your group therapy?

**Rules**

33.   What are your set group rules?

34.   Who developed them?

35.   How often do you review them with patients?

36.   How do you handle violations of the group rules?

37.   Do you have a treatment contract?

38.   What do you do about confidentiality?

39.   What are your criteria for giving patients more privileges?

40.   How much turnover is there?

41.   How often do people get discharged or referred out?

42.   How often do people choose to leave group and why?

43.   How do you handle returns to varying levels of relapse?

44.   How do you handle patients using other substances such as alcohol or marijuana?

**Outside of Group**

45.   Is there any care that goes on for these patients outside of group and what does that look like?

46.   Do you have higher levels of care you refer patients to?

47.   If so, what are those higher levels of care?

48.   Is there an intake visit with patients before group?

49.   Do you track any patient outcomes?

50.   If so, what are they?

51.   How often do you track them?

**Conclusion**

52.   Do you do anything to evaluate patients’ perspectives of group?

53.   Do you do anything to evaluate the person who runs group?

**54.**   **Is there anything that makes your group unique?**

55.   Is there anything you want to add that we have not talked about?
